# Ocular findings in patients with transfusion-dependent β-thalassemia in southern Iran

**DOI:** 10.1186/s12886-020-01647-y

**Published:** 2020-09-22

**Authors:** Sezaneh Haghpanah, Omid Reza Zekavat, Mohammadreza Bordbar, Mehran Karimi, Soheila Zareifar, Sanaz Safaei, Mani Ramzi, Hossein Ashraf

**Affiliations:** 1 Hematology Research Center, Shiraz University of Medical Sciences, Shiraz, Iran; 2 Poostchi Ophthalmology Research Center, Shiraz University of Medical Sciences, Shiraz, Iran

**Keywords:** β-Thalassemia, Iron-chelation therapy, Iron overload, Ocular manifestation, Retinal abnormality

## Abstract

**Background:**

Ocular involvement may occur via several mechanisms in patients with transfusion-dependent β-thalassemia (TDT) mainly chronic anemia, iron overload and iron chelator toxicity. We aimed to evaluate the frequency of abnormal ocular findings and their relationship with hematologic parameters in TDT patients.

**Methods:**

In this cross-sectional study from January 2018 to January 2019, a total of 79 patients with TDT over the age of 18 who were on iron-chelation therapy (ICT) were consecutively investigated. All patients were registered at the Thalassemia Comprehensive Center affiliated with Shiraz University of Medical Sciences, Shiraz, Southern Iran. Complete ophthalmic examination was performed by an expert ophthalmologist. Clinical and hematologic parameters were collected from the patients´ medical records.

**Results:**

The mean age ± standard deviation (SD) of the patients was 28.4 ± 5.6 years (range: 18–43). Twenty-four patients (30.4%) were male and 29 (36.7%) were splenectomized. The mean ± SD of the best-corrected visual acuity (VA) was 0.960 ± 0.086 decimal, (range: 0.6–1), 0.016 ± 0.046 logMar, (range: 0–0.2). The frequency of patients with VA > 0.1 logMar was 3 (3.8%). The mean intraocular pressure (IOP) was 14.88 ± 3.34 (6–25) mmHg. Fundus abnormalities were observed in 8 patients (10.1%), consisting of increased cup-disk ratio (3.8%), vessel tortuosity (2.5%), retinal pigment epithelium degeneration (2.5%), myelinated nerve fiber layer (1.3%), and internal limiting membrane wrinkling (1.3%). No significant association was observed between fundus abnormalities, VA, or IOP with hematologic parameters (*P* > 0.05). TDT patients with diabetes mellitus had significantly higher IOP (*P* = 0.010) but similar frequency of fundus abnormalities with non-diabetic patients (*P* > 0.05).

**Conclusions:**

The frequency of ocular abnormalities in our patients was lower than the previous reports. The frequency of fundus abnormalities were similar in diabetic and non-diabetic thalassemia patients indicating close monitoring and proper management of the disease and comorbidities in these patients.

## Background

Transfusion-dependent β-thalassemia (TDT) is the most common single-gene disorder worldwide. Mutations in the β-globin gene leads to reduction in β-globin chains and hemoglobin production; thus, hemolysis and ineffective erythropoiesis cause anemia. TDT patients incur iron overload due to the need for lifelong blood transfusion as well as red blood cell destruction that consequently leads to multi-organ dysfunction or failure. Although better medical care and regular iron chelation therapy (ICT) have led to better quality of life and increased in life expectancy, some adverse events might occur [1–3]. Ocular complications may occur via several mechanisms, such as microvasculature disease, chronic anemia, iron overload and iron chelator toxicity, as well as abnormal orbit growth due to unusual craniofacial growth [4, 5]. Common ophthalmologic findings in patients with TDT comprises of changes in visual acuity (VA), cataract and lens opacity, obliteration of iris pattern, shortened axial length, thickened lens, steepened corneal curvature, astigmatism, disturbances in tear function, and retinal abnormalities, such as retinal pigment epithelium (RPE) degeneration, optic neuropathy, retinal vessel tortuosity and vitreoretinal hemorrhage [4, 6–9].

In this study, we aimed to evaluate the frequency of ocular manifestations and their relationship with hematologic parameters in TDT patients.

## Methods

In this cross-sectional study from January 2018 to January 2019, a total of 79 patients with TDT over the age of 18 who were on ICT were consecutively investigated. All patients were registered at the Thalassemia Comprehensive Center affiliated with Shiraz University of Medical Sciences, Shiraz, Southern Iran. The study protocol was approved by the local Ethics Committee of Shiraz University of Medical Sciences (code = 11,886). After explaining the study objectives, written informed consent was obtained from all participants. TDT diagnosis was confirmed by hematologists based on clinical history and laboratory results, which included complete blood count and hemoglobin electrophoresis. TDT patients who were receiving regular blood transfusion with an interval of 2–4 weeks to maintain hemoglobin levels between 9 and 11 g/dl as well as receiving one type of ICT regimen within the last 2 years were included in the study. ICT was initiated for patients with serum ferritin levels above 1000 ng/ml at the time of diagnosis and it consisted of deferoxamine, deferasirox, or deferiprone with appropriate dosage either as monotherapy or combination therapy. Patients with other hemoglobinopathies or other types of anemia, those with any congenital ocular abnormality, history of ocular trauma and surgery or having no desire to participate in the study were excluded.

Complete ophthalmic examination was done for all patients by an expert ophthalmologist, including Snellen VA assessment by YANG vision tester (SIFI Diagnostic S.p.A., Treviso, Italy), refractive measurements including subjective and cycloplegic refractions, anterior segment examination by BM900 Haag-Streit slit lamp (Haag-Streit AG, Koniz, Switzerland) and motility examination. Intraocular pressure (IOP) was measured by two separate observers during afternoon hours using TONOREF II non-contact tonometry (NIDEK Co., Ltd., Gamagori, Japan) and the mean of the two readings were recorded. Cataract or any lens opacity was evaluated biomicroscopically, using BM900 Haag-Streit slit lamp and all opacities above grade 0 in the nuclear, cortical and/or subcapsular areas of the lens were reported [10]. Staining of the bulbar conjunctiva by fluorescein sodium and/or less than 10 s tear break up time (TBUT) were used as positive signs of dry eye. Posterior segment examination was done by indirect and biomicroscopic ophthalmoscopy, using Volk SuperField NC Lens (Volk Optical, Inc. Mentor, OH, USA).

In the statistical analyses related to IOP, VA, refractive error and cup-to-disk (CD) ratio, only measurements of the right eye was considered.

Clinical and hematologic parameters were collected from the patients´ medical records including serum ferritin and pre-transfusion hemoglobin levels, splenectomy status, type of ICT within the last 2 years, and any comorbidity.

### Statistical analysis

Data were analyzed by Statistical Package for the Social Sciences (SPSS Inc., Chicago, Illinois, USA) version 21. Shapiro-Wilk test was used to check the normality of quantitative variables. Descriptive data were presented as mean, standard deviation (SD), frequency and percentage. Comparison of the quantitative variables was performed by Student, t-test or Mann-Whitney test between the two groups and analysis of variance (ANOVA) test or Kruskal-Wallis test amongst more than 2 groups. Pearson and Spearman correlation coefficients were measured to determine the correlation of quantitative variables. A *P*-value less than 0.05 was considered to be statistically significant.

Sample size calculation: Considering α = 0.05, precision = 8%, and prevalence of ocular involvement = 85% [11], sample size was calculated as 77 patients using MedCalc software.

## Results

The mean age ± SD of the participants was 28.4 ± 5.6 years (range: 18–43). Demographic data, clinical characteristics, and ophthalmologic measurements of the study population are shown in Table 1. Twenty-four (30.4%) of the patients were male and 29 (36.7%) were splenectomized. The mean of serum ferritin and hemoglobin levels were 2245 ± 1623 (300–7500) ng/ml, and 9.8 ± 0.67 (8.1–11.1) g/dl, respectively. Serum ferritin level was ≤1000 ng/ml in 26.6%, between 1000 and 2000 ng/ml in 34.2%, and > 2000 in 39.2% of the patients. Thirty-six patients (45.5%) had comorbidities out of which diabetes mellitus (DM) was the most common (35.4%). With respect to the ICT regimens used in the past 2 years, the patients were categorized in four groups (Table 1). The most frequent ICT regimen was deferoxamine and deferiprone combination (34.2%) as well as deferasirox monotherapy (34.2%).

**Table 1:** Demographic and clinical characteristics and ophthalmologic measurements of patients with transfusion-dependent β-thalassemia

Variables	Values
**Age** (year)	28.4 ± 5.6 (18–43)
**Ferritin** (ng/ml)	2245 ± 1623 (300–7500)
**Weight** (kg)	52 ± 7.7 (36–85)
**Hemoglobin** (g/dl)	9.8 ± 0.67 (8.1–11.1)
**Splenectomy** (yes)	29 (36.7)
**Sex** (male)	24 (30.4)
**Comorbidities**^**a**^	36 (45.5)
Diabetes mellitus	28 (35.4)
Heart disease	2 (2.6)
Hypothyroidism	2 (2.6)
Hypoparathyroidism	1 (1.3)
Osteoporosis	3 (3.8)
Amenorrhea	1 (1.3)
**Type of iron-chelating therapy in the last 2 years**	
Deferoxamine	13 (16.5)
Deferasirox	27 (34.2)
Deferoxamine and Deferasirox	12 (15.2)
Deferoxamine and Deferiprone	27 (34.2)
**VA (decimal)**	0.960 ± 0.086
**VA (logMar)**	0.016 ± 0.046
0	69 (87.3)
0.1	7 (8.9)
> 0.1	3 (3.8)
**Intraocular pressure (**mmHg**)**	14.88 ± 3.34

*SD* standard deviation, *VA* visual acuity

^a^One patient had hypothyroidism and heart disease simultaneously

All values are presented as mean ± standard deviation for quantitative variables, and N (%) for categorical variables

The mean ± SD of the best-corrected VA in patients was 0.960 ± 0.086 decimal (range: 0.6–1), 0.016 ± 0.046 logMar, (range: 0–0.2). The mean spherical equivalent refractive error was − 1.24 ± 1.39 diopters (range: − 7.75 to 3.00). The frequency of patients with VA > 0.1 logMar was 3 (3.8%). Only one patient had anisometropic amblyopia in the left eye. None of patients had decreased best-corrected Snellen VA lower than 20/40. LogMar VA was positively correlated with age (logMar VA, r_s_ = 0.338, *P* = 0.002). Since the absolute value of the logMar VA changes in the opposite direction of VA, we can conclude that patients΄ VA negatively correlated with the patients’ age.

There was no significant association between VA and serum ferritin or hemoglobin levels, gender, splenectomy, or ICT (*P* > 0.05). The mean IOP was 14.88 ± 3.34 (6–25) mmHg. Only two patients (2.5%) had IOPs above 22 mmHg (24 and 25 mmHg). IOP did not significantly correlate with age, gender, hemoglobin or serum ferritin levels, splenectomy, or type of ICT (*P* > 0.05).

Positive findings of anterior and posterior ophthalmologic examinations of the patients are summarized in Table 2. The frequency of pinguecula and pterygium in the patients were 8.9% (95% confidence interval (CI): 3.6–17.4%) and 3.8% (95% CI: 0.8–10.7%), respectively. Overall, four patients (5.1%) had dry eye; one of them was using deferasirox, and three patients were using combined deferoxamine and deferiprone during the last 2 years. Of the four patients (5.1%) who had cataract or lens opacity, two patients were taking combination of deferoxamine and deferiprone, one patient was using combination of deferoxamine and deferasirox, and one was using deferoxamine monotherapy within the last 2 years.

**Table 2:** Frequency of abnormal ocular findings in patients with transfusion-dependent β-thalassemia

	N(%) confidence interval
**Conjunctiva**	
Nevus	3 (3.8) 0.8–10.7
Pinguecula	7 (8.9) 3.6–17.4
Pigmentation	6 (7.6) 2.8–15.8
**Cornea**	
Pterygium	3 (3.8) 0.8–10.7
Limbal girdle	4 (5.1) 1–12.5
**Motility dysfunction**	
Exophoria	17 (21.5) 13.1–32.2
Esophoria	6 (7.6) 2.8–15.8
**Dry eye**	4 (5.1) 1–12.5
**shallow Anterior Chamber**	3 (3.8) 0.8–10.7
**Cataract or lens opacity**	4 (5.1) 1–12.5
**Fundus abnormalities**	
large CD ratio (More than 30)	3 (3.8) 0.8–10.7
Vascular tortuosity	2 (2.5%) 0.7–8.77
RPE degeneration	2 (2.5%) 0.7–8.77
Myelinated NFL	1 (1.3) 0.2–6.9
ILM wrinkling	1 (1.3) 0.2–6.9

*CD* cup-to-disk, *RPE* retinal pigment epithelium, *NFL* nerve fiber layer, *ILM* internal limiting membrane

One patient had CD ratio more than 30 and ILM wrinkling simultaneously

Funduscopic examinations revealed fundus abnormalities in eight patients (10.1%) which consisted of large CD ratio in three patients (3.8%) and retinal abnormalities in six patients (7.6%), comprising vessel tortuosity (2.5%), RPE degeneration (2.5%), myelinated nerve fiber layer (NFL) (1.3%) and internal limiting membrane (ILM) wrinkling (1.3%). None of the three patients with large CD ratio had IOP higher than 18 mmHg. There was no significant association between funduscopic abnormalities and age, gender, hemoglobin or serum ferritin levels, splenectomy, or type of ICT (*P* > 0.05).

Clinical characteristics and ophthalmologic measurements were compared between the two groups of patients with and without DM (Table 3). Patients with DM had significantly higher age and splenectomy rate than patients without DM (*P* = 0.031 and *P* = 0.026 respectively). IOP was significantly higher in patients with DM in comparison with the patients without DM (16.17 ± 3.5 versus 14.17 ± 3.0, *P* = 0.010). Moreover, two diabetic patients had IOP > 22 mmHg (24 and 25 mmHg). Three patients (10.7%) from the DM group had VA > 0.1 logMar compared to zero in patients without DM (*P* = 0.041). The frequency of fundus abnormalities was comparable between diabetic and non-diabetic patients (*P* > 0. 999). The mean ± SD duration of DM in patients was 12 ± 4.7 (median: 12, range: 3–20) years. The patients with DM were divided into two groups based on the DM duration of less than 12 and ≥ 12 years. There were no significant differences between the two groups with respect to IOP, VA, the frequency of cataract and lens opacity, or the frequency of fundus abnormality (*P* > 0.05).

**Table 3:** Comparison of demographic and clinical characteristics between transfusion-dependent β-thalassemia patients with and without diabetes mellitus

Variables	Diabetes mellitus		*P*-value
	Yes = 28	No = 51	
**Age** (year) mean ± SD	30.2 ± 5.8	27.4 ± 5.2	0.031
**Hemoglobin** (g/dl) mean ± SD	9.9 ± 0.63	9.8 ± 0.69	0.636
**Ferritin** (ng/ml)	1855 ± 1325	2434 ± 1737	0.129
**Sex** (male) (%)	28.6	31.4	> 0.999
**Splenectomy** (yes) (%)	55.6	27.5	0.026
**Iron chelation therapy** (%)			
Deferoxamine	32.1	7.8	0.027
Deferasirox	21.4	41.2	
Deferoxamine and Deferiprone	35.7	33.3	
Deferoxamine and Deferasirox	10.7	17.6	
**Intraocular pressure (**mmHg) mean ± SD	16.17 ± 3.5	14.17 ± 3.05	0.010
**VA** (logMar) (%)			
0–0.1	89.3	100	0.041
> 0.1	10.7	0	
**Cataract or lens opacity**	10.7	2	0.125
**Fundus abnormalities** (%)	10.7	9.8	> 0.999

*VA* visual acuity

## Discussion

Thalassemia major is one of the most common hereditary disorders with a high prevalence in the Mediterranean region, such as Iran where there is a higher frequency of consanguineous marriage, a possible risk factor [12, 13]. Therapeutic measures have led to an increased life expectancy of the patients both in developing and developed countries. However, lifelong transfusion and iron overload can result in multi-organ dysfunction or failure. Furthermore, ICT may lead to some adverse events in these patients [14–16]. The frequency of ocular involvement in patients with thalassemia has already been evaluated in different populations. In this cross-sectional study, we described the frequency of ophthalmologic findings and their association with clinical characteristics and hematologic parameters in a group of Iranian patients with TDT. Overall, the frequency of ocular findings in our patients, both in anterior and posterior segment examinations were lower than what was reported in other parts of the world. Moreover, no significant relationship was found between ocular manifestation and clinical characteristics or hematologic parameters amongst patients with TDT.

Based on our results, none of our patients had reduced best-corrected Snellen VA lower than 20/40. In contrast, Taher et al. [6] and Gosai et al. [9] reported reduced vision with a frequency of 19.4 and 26% in younger patients compared with ours. Considering VA > 0.1 logMar in our study three patients (3.8%) had reduced vision compared to 23.2% of children with thalassemia major, which was reported by Aksoy et al. [7]. Jafari et al. [17] reported a frequency of 21.2% refractive errors in 54 patients with thalassemia major. Their patients were in a similar age range as ours, and the refractive errors were fully correctable with glass. Taneja et al. [4] also reported reduced VA in 33% of their patients, which refractive error was found to be the reason in all patients and was fully correctable with glasses. In our study, VA had only a significant correlation with age but not with serum hemoglobin, ferritin levels, splenectomy, or type of ICT. This is in line with Merchant et.al. study [18] who showed a significant correlation only with age and not with serum ferritin, ICT or blood transfusion requirement. Similarly, Taher et al. [6] found no significant relationship between reduced vision and ICT, which was contrary to some reports in the literature that revealed a likely correlation of deferoxamine and reduced VA [19, 20].

Intraocular pressure was in the normal range (< 22 mmHg) in almost all patients except two patients (24 and 25 mmHg). Similarly, IOP was reported to be in the normal range in patients with thalassemia major in other reports [17, 21]. However, they reported a significant increase in IOP compared to the values in healthy individuals. They suggested that it could be as a result of iron deposition in trabecular meshwork. In our study, IOP had no significant relationship with demographic or hematologic parameters, but the mean IOP was significantly higher in diabetic patients compared to non-diabetic ones. Additionally, the two detected patients with IOP > 22 mmHg were diabetic. Since DM is a common endocrinopathy in thalassemia patients [22] and due to the higher possibility of ocular involvement in diabetic patients, TDT patients with DM need more accurate ophthalmologic examinations and close monitoring. However, the frequency of fundus abnormalities in our study was similar in patients with and without DM. The higher frequency of reduced vision amongst diabetic patients in our study might be related to the older age range in this group. As it was mentioned, VA negatively correlated with age in our studied population.

The frequency of cataract and lens opacity in our study (5.1%) was less than the values that were reported in previous studies (6.3–45.7%) [5, 7, 11, 17]. In our study, all patients with cataract or lens opacity were treated with deferoxamine either as monotherapy or in combination with deferiprone or deferasirox for at least the past 2 years. Despite the likelihood of the relationship between cataract or lens opacity and different kinds of ICT which was previously reported in the literature about deferoxamine [4, 11], and deferiprone [23], the significance of this relationship was not documented in other studies [17, 21]. It seems that responsible mechanism might be oxidative stress and free radical damage as a result of iron overload. However, it should be kept in mind that patients with TDT usually change their ICT regimen during their lives while we only considered the ICT that was administered in the last 2 years in our patients with TDT.

The frequency of pterygium and pinguecula in our patients was 3.8% (95% CI: 0.8–10.7) and 8.9% (95% CI: 3.6–17.4) respectively compared to 0.5% (95% CI: 0.1–1) and 21% (95% CI: 18–24.1) in the similar age range of Iranian residents from a cross-sectional population-based study in Tehran. The occurrence of both disorders increases with age [24]. Other possible risk factors are diversity in climate and geographic variation, as closer to the equator the higher rates of these disorders become, which is possibly due to ultraviolet B light that causes cellular changes in the medial limbus of the cornea [25]. A higher rate of pterygium in patients with TDT in our study compared to what was reported in the similar age range of the normal Iranian population in the Tehran study can be explained by cellular changes as a result of iron overload and free radical damage.

In our study, six patients (7.9%) had retinal abnormalities in the form of non-pseudoxanthoma elasticum (PXE)-like manifestations in the posterior segment exam. The frequency of retinal abnormalities in our patients was lower than what was reported in previous studies [4, 6, 11, 17, 26]. Chronic anemia and iron overload are the major mechanisms responsible for retinal abnormalities in TDT patients [27, 28]. It seems that the appropriate management of chronic anemia with regular blood transfusion had been effective in our patients. However, approximately 70% of our patients had serum ferritin levels more than 1000 ng/ml and the presence of retinal abnormalities showed no significant association with serum ferritin levels, which could be due to the small study population with retinal abnormalities compared to the group without abnormalities. Moreover, we only used the serum ferritin level as a diagnostic marker for iron overload instead of the more accurate methods, such as heart and liver T2 magnetic resonance imaging or liver iron concentration measurement. Pseudoxanthoma Elasticum-like manifestations including angioid streaks, peau d’orange and optic disc drusen were not identified in our patients.

Based on our results, no significant relationship was detected between retinal abnormalities and type of ICT. There are some disputes in the literature regarding the relationship between ICT and the occurrence of ocular manifestations [4, 29–33]. Although ocular toxicity might be associated with administration of high-dose deferoxamine [34], it is usually reversible after drug interruption [35, 36]. For accurate assessment of iron chelating agents’ effect on the occurrence of ocular manifestations, a multicenter clinical trial study should be designed. However, it has several ethical concerns, for instance, it is not possible to stop ICT in one group.

Our study was limited due to the lack of a control group as well as the fact that maybe our patients had received other types of ICT prior to 2 years before this investigation. Consequently, there is a possibility that these ophthalmologic findings were related to the previously used ICTs. Moreover, due to small number of patients in subgroups of ocular manifestation, it was not possible to perform a statistical comparison based on the hematologic parameters of the TDT patients. For more accurate results, designing a longitudinal cohort study with baseline ophthalmologic examination in different ICT groups is warranted.

## Conclusions

According to the results, the frequency of ocular manifestations in our patients was lower than what was previously reported in other local and international studies. Moreover, no significant relationship between clinical findings and hematologic parameters was observed amongst our patients. The frequency of fundus abnormalities were similar in diabetic and non-diabetic thalassemia patients, indicating close monitoring and proper management of the disease and comorbidities in these patients. However, it should be taken into account that in this study, we only evaluated the clinical ocular findings. Further structural ophthalmic evaluation with more accurate methods such as optical coherence tomography and other multimodal imaging facilities is recommended. Also, due to the chronic nature of the disease, regular ophthalmologic assessment should be considered for early diagnosis of disease-related ocular complications as well as ICT toxicity.

## Data Availability

The methodology is listed in detail in the methods section of the manuscript. All data are available upon request.

## Abbreviations

TDT: Transfusion-dependent β-thalassemia
ICT: Iron-chelation therapy
SD: Standard deviation
VA: Visual acuity
IOP: Intraocular pressure
RPE: Retinal pigment epithelium
CD: Cup-to-disk
SPSS: Statistical Package for the Social Sciences
ANOVA: Analysis of variance
DM: Diabetes mellitus
CI: Confidence interval
NFL: Nerve fiber layer
ILM: Internal limiting membrane
PXE: Pseudoxanthoma elasticum
